# Multifunctional, Robust, and Porous PHBV—GO/MXene Composite Membranes with Good Hydrophilicity, Antibacterial Activity, and Platelet Adsorption Performance

**DOI:** 10.3390/polym13213748

**Published:** 2021-10-29

**Authors:** Yuandong Wu, Weishuang Zheng, Yinan Xiao, Beining Du, Xingru Zhang, Min Wen, Chen Lai, Yi Huang, Liyuan Sheng

**Affiliations:** 1 Shenzhen Institute, Peking University, Shenzhen 518057, China; wuyd@ier.org.cn (Y.W.); zhengws@ier.org.cn (W.Z.); xiaoyn@ier.org.cn (Y.X.); bndu10s@alum.imr.ac.cn (B.D.); zhangxr@ier.org.cn (X.Z.); min.wen@siat.ac.cn (M.W.); laic@ier.org.cn (C.L.); yhuang@pku.edu.cn (Y.H.); 2 PKU-HKUST Shenzhen-Hong Kong Institution, Shenzhen 518057, China; 3 School of Environmental Science and Engineering, Peking University, Beijing 100871, China

**Keywords:** MXene, PHBV, composite membrane, hydrophilicity, antibacterial properties, coagulation

## Abstract

The limitations of hydrophilicity, strength, antibacterial activity adsorption performance of the biobased and biocompatible polymer materials, such as polyhydroxyalkanoates (PHAs), significantly restrict their wider applications especially in medical areas. In this paper, a novel composite membrane with high antibacterial activity and platelet adsorption performance was prepared based on graphene oxide (GO), MXene and 3-hydroxybutyrate-co-hydroxyvalerate (PHBV), which are medium-chain-length-copolymers of PHA. The GO/MXene nanosheets can uniformly inset on the surface of PHBV fibre and give the PHBV—GO/MXene composite membranes superior hydrophilicity due to the presence of hydroxyl groups and terminal oxygen on the surface of nanosheets, which further provides the functional site for the free radical polymerization of ester bonds between GO/MXene and PHBV. As a result, the tensile strength, platelet adsorption, and blood coagulation time of the PHBV—GO/MXene composite membranes were remarkably increased compared with those of the pure PHBV membranes. The antibacterial rate of the PHBV—GO/MXene composite membranes against gram-positive and gram-negative bacteria can reach 97% due to the antibacterial nature of MXene. The improved strength, hydrophilicity, antibacterial activity and platelet adsorption performance suggest that PHBV—GO/MXene composite membranes might be ideal candidates for multifunctional materials for haemostatic applications.

## 1. Introduction

All countries are aware of the serious environmental problems caused by white pollution with the rapid development of the national economy, especially the petrochemical industry [1,2,3,4]. Every year, the amount of plastic waste that is difficult to recycle reaches tens of millions of tons [5,6]. China has already committed to achieving peak emissions of carbon dioxide before 2030 and carbon neutrality before 2060 [7,8]. The government urged quicker steps to take actions that enabled the peak of emissions [9]. It is necessary that the government accelerates bolstering the development of the green industry [10,11]. Hence, the widespread use of biodegradable materials is expected.

At present, biodegradable materials such as (1) natural polymer materials derived from dechitosan, cellulose, and starch [12,13,14]; (2) synthetic materials such as poly (glycolic acid) (PGA), poly (lactic acid) (PLA), poly (butylene succinate) (PBS), copolymer of aliphatic polyester, and polyamide (CPAE) [15,16,17]; and (3) microbial synthetic materials such as polyhydroxyalkanoate (PHAs) [18,19,20] have attracted widespread attention. Due to its good biocompatible and biodegradable properties [21,22], polyhydroxyalkanoate (PHAs) generated by bacteria growing under oligotrophic conditions is considered a promising haemostatic material and has attracted extensive attention from medical biomaterial researchers [21,22,23].

PHA is a kind of thermoplastic polyester synthesized by prokaryotes as a carbon source and energy storage under the carbon and nitrogen imbalance condition [24,25]. Poly(3-hydroxybutyrate) (PHB), discovered by M. Lemoigne in 1925, was the first-generation product in the PHAs family [26,27]. At least 150 different monomer structures of PHA have been discovered to date [28,29,30,31,32]. The medium-chain-length-copolymers poly(3-hydroxybutyrate-co-hydroxyvalerate) (PHBV) and poly(3-hydroxybutyrate-co-hydro-xyhexanoate) (PHBHHx) are the latest generation products in the PHAs family, with improved toughness and processability. However, the tensile strength and adsorption performance of PHBV and PHBHHx still need to be further improved to achieve the application requirements of haemostatic materials [21]. Moreover, fewer functional features, such as poor antibacterial and hydrophilic capacity, further limit PHAs applications, especially in biomedical materials [4,21]. PHAs-based materials have been widely applied in the fields of biomedicine, such as biodegradable sutures, bone tissue engineering and drug-loaded applications [21,33,34,35]. However, few investigators studied PHAs-based materials to simultaneously solve the problems of surgical wound haemostasis. Vigneswari et al. prepared nanofibrous P(3HB-Co-4HB)/collagen peptide constructs using an electrospinning system and the results indicate that these PHAs-based composites may be a potential leave-on wound dressing but have insufficient antimicrobial activity and hydrophilic capacity [36].

In recent years, these problems have led to a new breakthrough with the rapid development of nanotechnology. Graphene oxide (GO) is a novel two-dimensional material with high hydrophilicity and excellent mechanical performance and has considerable good potential for preparing high-performance polymer nanocomposites [4,37,38]. Abundance of oxygenic groups of GO can provide binding sites with organic polymer matrices, such as PHAs [4]. The poor dispersibility makes the GO sheets agglomerate and results in poor adhesion between GO and PVAs, which gives polyvinyl acetate and GO (PVA—GO) composites good tensile strength but low breaking elongation [39]. Some investigators further improved the dispersion of GO in polymers through chemical and physical modifications [4,40]. Niyogl et al. found that GO modified via alkylamine can give GO good dispersion in organic solutions [41]. The ball milling of GO can improve the dispersibility in PHBV and develop PHBV—graphene nanocomposites with good tensile properties [42]. Xu et al. fabricated PHA films with good tensile and antibacterial activity by grafting them with functionalized GO [4]. These studies confirmed that GO could be loaded in as a polymer as nanofiller to improve the mechanical performance of organic polymers, which is critical to improving the performance of polymers and GO composites. However, few investigations have discussed the prospects of GO as a multifunctional nanofiller to simultaneously enhance the tensile, hydrophilic, and antimicrobial properties of PHAs for haemostatic materials.

Mxenes, first discovered by Naguib et al. in 2011 [43], are a developing novel material family of two-dimensional (2D) materials with a formula of M_n+1_X_n_T_x_, which are composed of transition metals (M), carbides/nitrides (X), and functional groups such as hydroxyl, oxygen or fluorine (X) [44,45,46]. The presence of hydroxyl groups or terminal oxygen on the surface gives MXene materials more application prospects, such as high hydrophilicity and processability [47]. The laminar structure and hydrophilicity give MXene materials rapid and selective delivery of water molecules. Many investigations also revealed that MXene materials obtain high permeable flux. In addition, some investigators have confirmed that MXene nanosheets have antibacterial properties and that the sharp edges of MXene nanosheets lead to the release of bacterial DNA [48].

This study attempts to describe the development and detection of a novel PHBV—GO/MXene composite membrane with improved mechanical properties, antibacterial abilities, high hydrophilicity, and adsorption capacity. These characteristics make the PHBV—GO/MXene composite membrane an ideal material for biodegradable haemostatic applications. Various contents of GO and MXene have been blended with PHBV to prepare PHBV—GO/MXene composite membranes by electrostatic spinning techniques. The mechanical properties, microscopic morphology, physical properties, antibacterial properties, and adsorption properties of the composites were analysed. Therefore, the investigation aimed to provide a biodegradable haemostatic material with good mechanical properties, excellent antibacterial abilities, high hydrophilicity, and adsorption capacity.

## 2. Materials and Methods

### 2.1. Materials

PHBV containing 92.3 mol% 3-hydroxybutyrate and 7.7 mol% hydroxyvalerate was bacterially synthesized. Ti_3_AlC_2_ MAX powders, graphite, methylene chloride (DCM), sulfuric acid (H_2_SO_4_), hydrochloric acid (HCl), potassium permanganate (KMnO_4_), 40% HF, hydrogen peroxide (H_2_O_2_), and lithium fluoride (LiF) were obtained from Shanghai Aladdin Bio-Chem Corporation (Shanghai, China). Deionized (DI) water was produced by a Milli-Q HX 7040 water system (Burlington, MA, USA). All reagents without special instructions were of analytical grade.

### 2.2. Synthesis of Graphene Oxide (GO) and MXene (Ti_3_C_2_T_x_)

The preparation method of GO used the Hummers’ method [49].

MXenes are mainly synthesized from MAX precursors. Two grams of Ti_3_AlC_2_ MAX phase powder was slowly added to 2 g LiF, and 20 mL 9 M HCl [37] and stirred by magnetic stirrers for 24 h at 35 °C. The products were washed until the pH values of the solutions reached 6, using DI water with centrifugation at 3500 rpm for 5 min per cycle. The sediment was Ti_3_CT_x_ and needed to be shaken vigorously for approximately 10 min. Finally, the unexfoliated sheets were removed via centrifugation at 3500 rpm for 1 h. Then, the concentration of MXene solution reached 1 mg/mL.

### 2.3. Electrospinning of Composite Membranes

**Figure 1:**
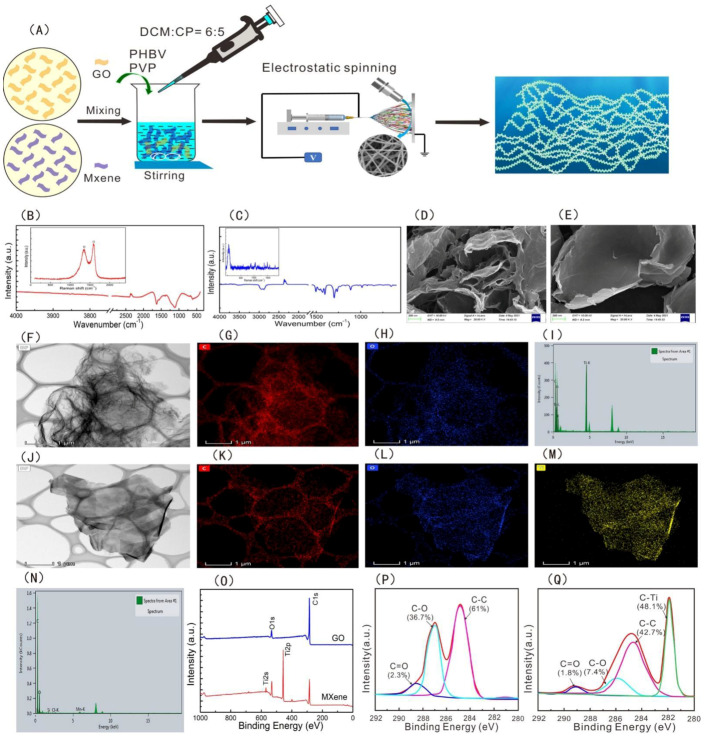
Schematic diagram of preparation of the PHBV—GO/MXene composite membranes (**A**), FT-IR and Raman spectra of GO (**B**), and MXene (**C**); SEM images of GO (**D**) and MXene (**E**). TEM analysis of GO (**F**) and MXene (**J**). Distribution of elements: C for GO (**G**), O for GO (**H**), C for MXene (**K**), O for MXene (**L**), and C for MXene (**M**). The element composition of GO (**N**) and MXene (**I**). XPS spectra of GO and MXene (**O**). C1s XPS spectra of GO (**P**) and MXene (**Q**).

PVP, a water-soluble polymer, was added before electrospinning to promote pore formation to avoid the swathing of GO and MXene nanoparticles in PHBV fibre mats. PHBV and PVP were dissolved in solvents containing DCM and acetone (6:5 *v:v*) along with 0.5% and 1% nanoparticles with a GO/MXene mass ratio of a 1/4 to prepare porous electrospun fibres. Some investigations suggested that the composite membrane with a 1/4 GO/MXene ratio had good water flux [37], which may help improve the hydrophilicity and adsorption capacity of PHBV—GO/MXene composite membranes. The weight percentage of PHBV and PVP was 5:1. The mixed solution was magnetically stirred for 18 h and then a 6 mL syringe filled with homogenous polymer solutions was connected to a needle. The electrospinning conditions were 25 kV voltage, 0.1 mL/h speed and 15 cm width between two parallel plates. The electrospinning process continued for 10 h at 25 °C and 60% relative humidity. After electrospinning, PVP was removed by washing it with water at 70 °C and ultrasonic treatment. Finally, the washed fibre mats were dried in a vacuum (Figure 1A).

### 2.4. Characterization Methods

The surface morphologies of the fibre mats were examined by field emission scanning electron microscopy (FE-SEM, Merlin, Berlin, Germany). Transmission electron microscopy (TEM, JEOL Ltd., Tokyo, Japan), Alpha 300M Raman spectrometry (WITec, Berlin, Germany), energy spectroscopy (EDS, Oxford, London, UK), BRUKER D8 Advance (Berlin, Germany), Frontier FT-IR Spectrometer (Berlin, Germany), Thermo Fisher Scientific K-Alpha (Waltham, MA, USA), and DSA100 (Berlin, Germany) were used to obtain insight into the microstructure and composition of PHBV—GO/MXene composite membranes.

### 2.5. Platelet Adsorption Experiments

The haemostatic performance of the PHBV and PHBV—GO/MXene composite membranes was evaluated by platelet adsorption and blood coagulation. The blood of healthy rabbit with 3.8% anticoagulant sodium citrate (2:8) was used to make platelet-rich plasma (PRP). A Thermo Scientific centrifuge was used to centrifuge with 1500 rpm at 4 °C and hold 10 min. 5 cm^2^ PHBV and PHBV—GO/MXene composite membranes were dropped in 5 mL of PRP at 37 °C. Platelets were detected before and after adsorption using platelet analyzer. Three parallel experiments were carried out. Blood coagulation time was detected using the method of Wu et al. (2013) [50].

### 2.6. Method of the Antibacterial Activity Test

The antibacterial experiment of PHBV—MXene composite membranes were divided into PHBV, PHBV—GO/MXene 0.1%, PHBV—GO/MXene 0.5%, PHBV—GO/MXene 1%, and the blank group against *E. coli* and *S. aureus.* The composition of luria broth (LB) solid medium is: 10 g/L peptone, 10 g/L NaCl, 5.0 g/L beef extract, and 20% agar powder. The dosages of PHBV, PHBV—GO/MXene 0.1%, PHBV—GO/MXene 0.5%, and PHBV—GO/MXene 1% composite membranes were 10 mg. The bacteria used here were *E. coli* (ATCC 25922) and *S. aureus* (ATCC 6538). The liquid LB medium was used for cultures *E. coli* and *S. aureus* at 37 ° C and 200 rpm. Bacteria growth kinetics was measured using the optical density (OD) of bacteria suspensions at 600 nm. Bacteria with 0.1 OD_600_ (10^8^CFU/mL), which indicate the *E. coli and S. aureus* were in an exponential phase, were used to perform thentibacterial activity test. A measured 10 mg composite membranes and 10μL bacterial solution were evenly smeared on the surface of solid LB medium. Then culture dishes with bacteria and composite membranes were cultivated for 24 h at 37 °C in a microbiological incubator. The camera took pictures of the culture dishes after culture and identified the number of bacteria colony.

The antibacterial activity against *E. coli* (α) and *S. aureus* (β) of PHBV and PHBV—GO/MXene composite membranes were calculated using Equations (1) and (2), respectively.

(1) $$\alpha =\frac{C_{t}}{C_{0}}\times 100\%\;$$

(2) $$\beta =\frac{D_{t}}{D_{0}}\times 100\%\;$$

where $C_{0}$ and $C_{t}$ are the colony numbers of *E. coli* in the blank group and experimental groups, respectively. $D_{t}$ represents the colony numbers of *S. aureus* in experimental groups, and $D_{0}$ represents the colony numbers of *S. aureus* in the blank group.

## 3. Results and Discussion

### 3.1. Characterization of GO and MXene Nanosheets

To observe the microscopic morphology characteristics of GO and MXene nanosheets, SEM and TEM were used. The microscopic morphology characteristics of the GO and MXene nanosheets are illustrated in Figure 1. The FT-IR spectral bands of GO showed characteristic peaks of C=O, and C–O–C stretching vibrations, which occur at 1715 cm^−1^ and 1045 cm^−1^. (Figure 1B). The FT-IR spectra of MXene nanosheets appeared at 3423 cm^−1^, 2923 cm^−1^, 2848 cm^−1^, 1715 cm^−1^, and 1045 cm^−1^, which correspond to the –OH, C-H in benzene rings and alkane carbon chains, C=O, and C–O–C stretching vibration, respectively [37], mainly owing to the oxygenic groups on GO and MXene. GO had Raman characteristic peaks at 1365 cm^−1^ and 1610 cm^−1^, belonging to the D peak and G peaks of carbon atomic crystals. The significance of the D peaks confirms the effective introduction of oxygenic groups into GO; the G peak is the stretching vibration of the graphene lattice [37]. Raman spectral bands appeared at 201 cm^−1^ and 720 cm^−1^, which are the characteristic Raman peaks of MXene nanosheets [51].

The SEM images showed that the morphologies of the GO and MXene nanosheets are lamellar textures (Figure 1C,D), and the TEM images also indicated that the textures of GO and MXene nanosheets are lamellar (Figure 1F,J). X-ray photoelectron spectroscopy (XPS) and energy dispersive spectrometry (EDS) mapping illustrated that GO nanosheets are mainly composed of C and O elements, and MXene nanosheets contain C, O, and Ti elements (Figure 1K,L,N,O). The C1s XPS spectra further showed that GO was fitted into three peaks (C=O, C-O, and C-C) and that MXene had four peaks (C=O, C-O, C-C, and C-Ti) (Figure 1P,Q). The results suggested that there are plenty of hydrophilic groups, for example, hydroxyl and carboxyl groups, located on nanosheets of GO and MXenen surfaces. These factors may be helpful for improving the hydrophilic performance of PHBV.

### 3.2. Characterization of Composite Membranes

The microscopic morphology characteristics of the PHBV and PHBV—GO/MXene composite membranes were remarkably affected by the different GO/MXene dosages (Figure 1). The PHBV fibre had a smooth surface with many nanopores (Figure 1A,E). Elements of C and O are the main components (Figure 1A). So many three–dimensional (3D) nano-round pores may indicate that the PHBV—GO/MXene 0.1% composite membranes have good mechanical properties, because round holes can avoid stress concentrations (Figure 1B). The main elements of the PHBV—GO/MXene 0.1% composite membranes were still C and O, but the energy dispersive X-ray spectrometry (EDS) results showed that a certain amount of Ti was introduced by MXene (Figure 1B). PHBV—GO/MXene 0.5% composite membrane morphology is similar to that of PHBV (Figure 1A, C), but the elemental compositions of the PHBV—GO/MXene 0.5% composite membranes were C, O, and Ti (Figure 1C), similar to PHBV—GO/MXene 0.1% composite membranes (Figure 1B). A large number of fusiform structures and nanofibres with small diameters appearing simultaneously may indicate that the mechanical properties of PHBV—GO/MXene 1% composite membranes were poor (Figure. 1D). The fusiform structures may be caused by abnormal turbulence during electrospinning due to the increase in solution viscosity [52]. Many Ti elements are contained in the PHBV—GO/MXene 1% composite membranes (Figure 1D). The dark patches on the surface of the fibres with 0.1% and 0.5% GO/MXene content likely indicate GO/MXene aggregation (Figure 1F,G). However, no dark patches on the surface of the fibres further suggested that the fusiform structures are the aggregate of GO/MXene in the PHBV—GO/MXene 1% composite membranes (Figure 1H).

**Figure 2:**
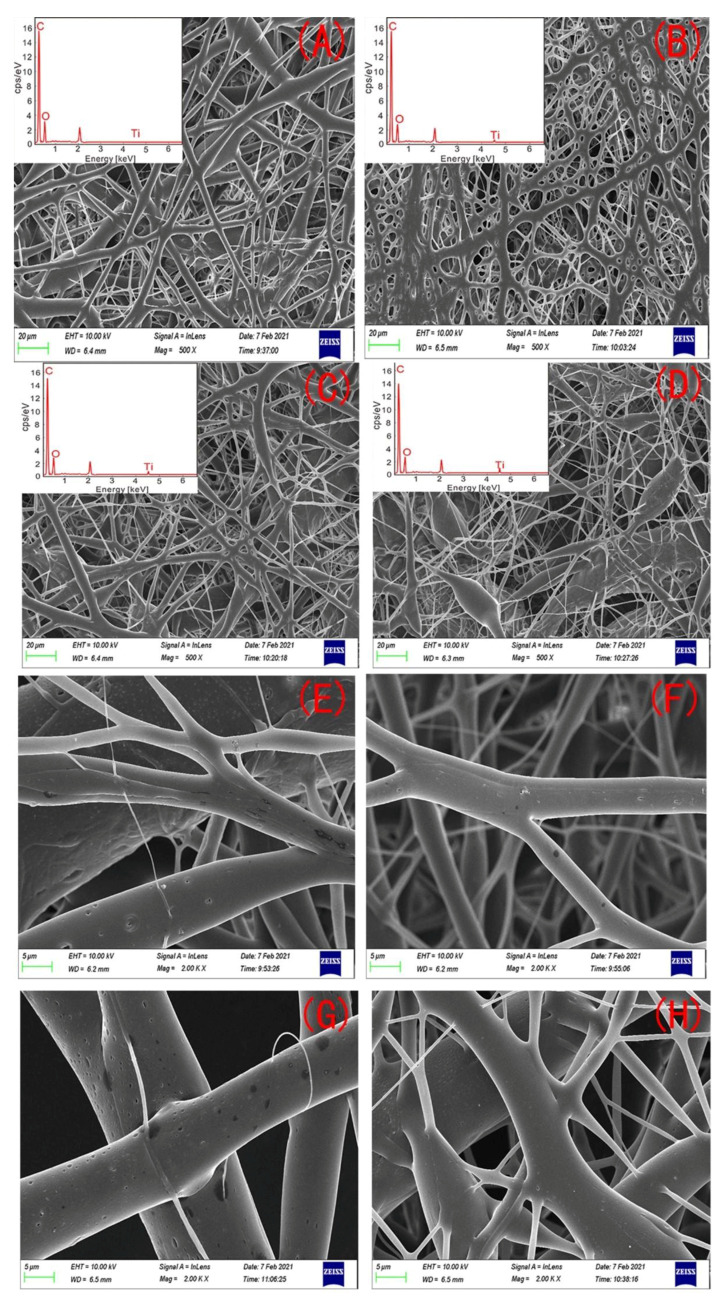
SEM images of PHBV (**A**,**E**), PHBV—GO/MXene 0.1% (**B**,**F**), PHBV—GO/MXene 0.5% (**C**,**G**), and PHBV—GO/MXene 1% (**D**,**H**) composite membranes. Inset figures show the EDS spectrum.

The PHBV fibres had a diameter of 4.32 ± 1.93 μm (Figure 2A,E). Adding GO/MXene resulted in feature surface morphology (Figure 2B–D,F–H). PHBV—GO/MXene 0.1%, PHBV—GO/MXene 0.5%, and PHBV—GO/MXene 1% had diameters of 3.23 ± 2.06 μm, 3.16 ± 2.43 μm, and 1.36 ± 2.06 μm, respectively (Table 1). The combined weight percentages of Ti in the PHBV—GO/MXene 0.1%, PHBV—GO/MXene 0.5%, and PHBV—GO/MXene 1% fibre mats were 0.07%, 0.28%, and 0.75%, respectively (Table 1). These weight percentages detected were lower than that of Ti addition. This may be due to the loss of mass in the fabrication process of the fibre mats and/or instrumental error.

**Figure 3:**
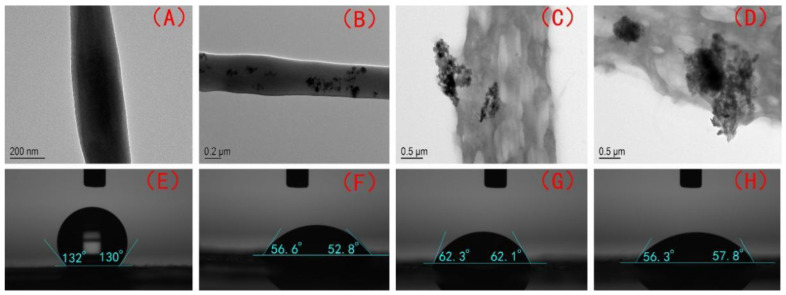
TEM images and water contact angle of PHBV (**A**,**E**), PHBV—GO/MXene 0.1% (**B**,**F**) PHBV—GO/MXene 0.5% (**C**,**G**), and PHBV—GO/MXene 1% (**D**,**H**) composite membranes.

The morphologies of the PHBV and PHBV—GO/MXene composite membranes were also detected by TEM. Figure 3A−D shows the microscopic morphology characteristics of single PHBV and PHBV—GO/MXene fibres. Figure 3A presents the smooth surface of the PHBV fibre, while the surface of the PHBV—GO/MXene fibre has many dark patches (Figure 3B–D). The smooth surfaces of the PHBV and PHBV—GO/MXene fibres are consistent with the SEM observations (Figure 2F,G). These nano-GO/MXene embedded in the surface of the fibre nanosheets may act as active hydrophilic sites. The surface of PHBV fibres may help absorb platelets and accelerate blood coagulation for the hydrophobicity of PHBV [53,54,55].

The water contact angles (130.0 ± 2.3°, Table 1) of the PHBV composite membranes were similar to those reported for EPF mats [56] and PVDF membranes [52]. They were also lower than those of hydrophobic ZnO-containing fibre mats (152 ± 3.0°) [57] and SiO_2_-containing fibre mats (160.5 ± 2.3°) [58,59] but higher than those of PHBV—GO/MXene membranes (Table 1). The water contact angles of the PHBV—GO/MXene 0.1%, PHBV—GO/MXene 0.5%, and PHBV—GO/MXene 1% composite membranes were 54.2 ± 3.1°, 62.2 ± 1.8°, 55.9 ± 2.8°, respectively (Table 1; Figure 3E–H). The water contact angles of the PHBV—GO/MXene composite membranes were not significantly different in present investigation. Many investigators also found similar results, where nanoparticles dosage did not dramatically change the water contact angle [56,60,61].

The better hydrophilicity of PHBV—GO/MXene composite membranes is due to the high amount of hydrophilic functional groups, as hydroxyl and carboxyl groups [37,62]. Moreover, the laminar flow structure of MXene nanosheets can rapidly and selectively transport water molecules [37,45], which further improves the hydrophilicity of PHBV—GO/MXene composite membranes. In addition, some investigations have also reported that increasing the interlayer spacing and introducing nanoparticles into MXene can further improve the water flux and antifouling properties [63,64]. Therefore, the interlayer spacing of MXene and the doping effect of GO and MXene are other reasons to enhance the hydrophilicity of PHBV—GO/MXene composite membranes.

**Figure 4:**
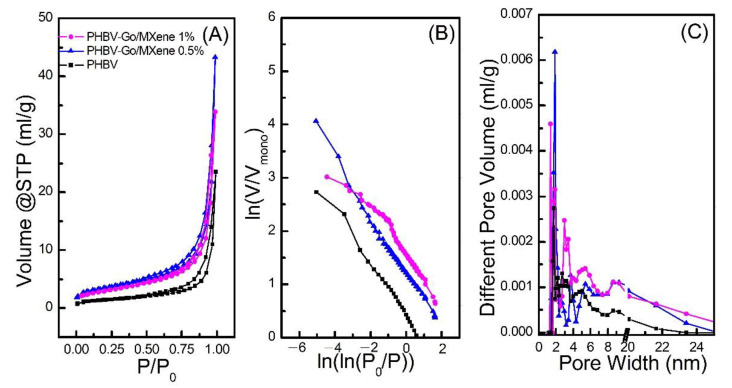
Plots of N2 adsorption—desorption (**A**), ln (V/Vmono) vs. ln (P/P_o_) (**B**) and pore diameter (**C**) distributions of PHBV, PHBV—GO/MXene 0.5%, and PHBV—GO/MXene 1% composite membranes.

BET-SA and PSD analyses were carried out to investigate the adsorption and pore characteristics of PHBV and PHBV—GO/MXene composite membranes. The results are illustrated in Figure 4. The isotherms of PHBV and PHBV—GO/MXene composite membranes showed hysteresis loops formed by the separation of adsorption—desorption curves (Figure 4A). Generally, a hysteresis loop occurs in the mesoporous materials [65,66]. Based on the thermodynamics principle, the formation of a hysteresis loop is related to the difference in heat released and absorbed during the adsorption—desorption process [67,68]. According to the IUPAC classification criteria, the isotherms of the PHBV and PHBV—GO/MXene composite membranes were type I (Figure 4A), indicating a highly uniform pore size, good pore connectivity, rapid coagulation, and monolayer adsorption. Figure 4 also shows that the adsorbed N_2_ increased after the P/P_o_ rate increased from 0 to 0.9. Moreover, the highest separation of adsorption—desorption curves appeared at P/P_o_ = 0.9, which confirms that many micropores consist of PHBV and PHBV—GO/MXene composite membranes. In addition, the more remarkable hysteresis loop further suggests that the proportion of micropores is higher in the PHBV—GO/MXene composite membrane than that in the PHBV composite membrane.

The pure PHBV membranes had a 16.75 m^2^ g^−1^ surface area (SA) and 0.023 cm^−3^ g^−1^ pore volume (PV) (Table 1; Figure 4A) and the peak N_2_ adsorption value was 23.52 cm^−3^ g^−1^ at P/P_o_ = 0.99. Notably, 45.51 m^2^ g^−1^ of SA, 0.051 cm^−3^ g^−1^ of PV and 43.23 cm^−3^ g^−1^ of the maximum N_2_ adsorption capacity indicate that the adsorption capacity of PHBV—GO/MXene 0.5% composite membranes was higher than that of PHBV—GO/MXene 1% composite membrane, which had 24.46 m^2^ g^−1^ of SA, 0.035 cm^−3^ g^−1^ of PV and 33.89 cm^−3^ g^−1^ of the maximum N_2_ adsorption capacity (Table 1; Figure 4A).

Furthermore, fractal dimension (D) was calculated to investigate the characteristics of pore structure in PHBV and PHBV—GO/MXene composite membrane, based on the Frenkel—Halsey—Hill (FHH) theory and the isotherms of the PHBV and PHBV—GO/MXene composite membrane (Figure 4B). The D value of the PHBV—GO/MXene 0.5% composite membrane was the maximum observed for the plots of ln (V/V_mono_) vs. ln(ln(P_o_/P)), however, the low plot range of ln (V/V_mono_) vs. ln(ln(P_o_/P)) indicate that the pure PHBV composite membrane had the lowest D value. The D values of the PHBV—GO/MXene 0.5% composite membrane were similar to those of the pure PHBV composite membrane. The calculated D values of the PHBV, PHBV—GO/MXene 0.5%, and PHBV—GO/MXene 1% composite membranes were 2.371, 2.489, and 2.563, respectively, which further confirmed that the PHBV—GO/MXene composite membranes were structurally different and mainly contained mesopores and micropores. Figure 4C shows that most mesopores on the surface of the PHBV, PHBV—GO/MXene 0.5%, and PHBV—GO/MXene 1% composite membranes are 1–6 nm in diameter. The lowest amounts of pores with diameters lager than 6 nm confirm the higher proportion of micropores in the PHBV composite membrane. In addition, the 3D hierarchical architectures of the PHBV and PHBV—GO/MXene composite membranes can provide active sites and may act as transmission paths that can accelerate the adsorption process.

**Figure 5:**
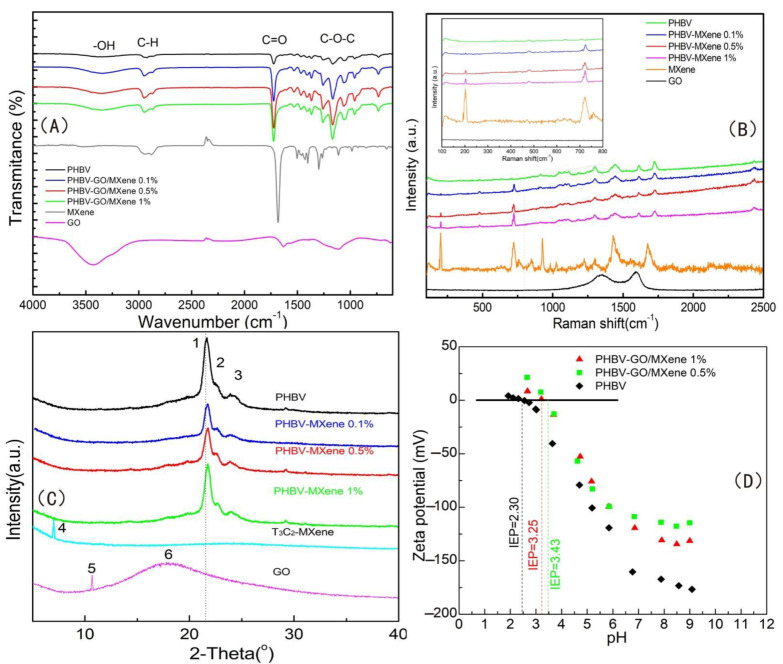
FT-IR (**A**), Raman (**B**), and XRD (**C**) spectra of PHBV, GO, MXene, PHBV—GO/MXene 0.1%, PHBV—GO/MXene 0.5%, and PHBV—GO/MXene 1% composite membranes. Zeta potential (**D**) of PHBV, PHBV—GO/MXene 0.5%, and PHBV—GO/MXene 1% composite membranes.

Fourier transform infrared spectroscopy, Waltham, MA, USA), Raman spectroscopy (Berlin, Germany), X-ray diffraction (XRD) spectra (Waltham, MA, USA) and zeta potential (ξ) (Waltham, MA, USA) values of the PHBV and PHBV—GO/MXene composite membranes were also obtained to investigate the surface chemistry of the composite membrane. Moreover, FT-IR, Raman, and XRD analyses of GO and MXene were performed as references. The results are illustrated in Figure 5.

The peak at ~3390 cm^−1^ in the FT-IR spectrum of PHBV can be assigned to the stretching vibration of the hydroxyl (-OH) group (Figure 5A). The peaks appearing at 2921 and 2857 cm^−1^ corresponded to the C-H stretching vibrations. The peaks at 1723 and 1050 cm^−1^ can be assigned to the C=O stretching vibration and the C–O–C stretching vibration, respectively [4]. For pure GO, the broad band at ~3423 cm^−1^ corresponded to the stretching vibration of hydroxyl –OH. The peaks at 2923 and 2848 cm^−1^ corresponded to the C-H stretching vibrations (Figure 5A). The peaks at 1715 and 1045 cm^−1^ corresponded to the C=O stretching vibration and the C–O–C stretching vibration, respectively [37]. MXene nanosheets had strong C=O stretching vibrations in the carboxyl group, while the C–O–C stretching vibrations were weak (Figure 5A). The stretching vibration of –OH, C=O, and C–O–C increased with the mixing of GO/MXene into the PHBV—GO/MXene composite membrane, due to the oxygenic groups on the surface of GO and MXene.

Usually, the Raman spectral bands of GO showed two broad peaks between 1000 and 1800 cm^−1^. The peak appearing at high wavenumbers was the G-band, which usually appeared between 1500 and 1600 cm^−1^. The peak appearing at low wavenumbers was the D-band, which was generally located between 1300 and 1400 cm^−1^. The G-band is induced by crystalline graphitic/*sp*^2^ carbon atoms and involves out-of-phase intra-layer displacement in the graphene structure; the latter originates from *sp^3^* hybridization and relates to polycrystalline imperfect graphite [66,69]. The peaks at 201 and 720 cm^−1^ are the characteristic peaks of MXene [37,51]. Among them, the 201 cm^−1^ peak was the symmetric out-of-plane vibration (A1g) of Ti and the 720 cm^−1^ peak was the out-of-plane vibration (A1g) of C [51]. The peak range from 230 to 470 cm^−1^ corresponded to the in-plane (Eg) vibration of the surface groups around titanium atoms [51]. The peaks at 201 and 720 cm^−1^ identifying MXene were more evident with increasing GO/MXene dosage in PHBV—GO/MXene composite membrane (Figure 5B). These results indicate that GO/MXene is effectively mixed into PHBV, which is consistent with the SEM and TEM results (Figure 3 and Figure 4).

The XRD pattern of GO shows a broad band at 18.2° and a sharp peak at 10.7°, which are the characteristic peaks of GO [37]. The small peak at 7° is the characteristic peak of MXene [37]. In addition, the 7° peak also indicates that the sample has not been oxidized by air and is well preserved [70]. The peaks of 2θ appearing at 21°, 22°, and 26° suggest that there were helical lamellae in the pure PHBV membrane. The crystal planes were (101), (111), and (121). The lower diffraction peaks of PHBV—GO/MXene may indicate that PHBV has a smaller crystal size (Figure 5C), which further suggests that GO/MXene modification may increase the molecular structure of the polymer and reduce the crystal forming ability of the PHBV molecule. The lower diffraction peaks also indicate that the grain size of PHBV in the PHBV—GO/MXene membrane is smaller than that in PHBV (Figure 5C). Therefore, the decrease in the crystallization index (CI) may suggest a significant improvement in the toughness of the PHBV—GO/MXene membrane due to the GO/MXene hybrid effect. In addition, the XRD peaks of the PHBV—GO/MXene membrane are shifted at high angles, which may indicate that GO/MXene enters the PHBV crystal. The higher diffraction peak of the PHBV—GO/MXene 1% composite membrane may be due to the agglomeration effect, resulting in a small amount of GO/MXene in the PHBV crystal, which has a high angle deflection and a strong diffraction peak.

The zeta potential (ξ) values of the PHBV and PHBV—GO/MXene composite membranes were detected within a pH range from 2 to 11 (Figure 5D). The ζ values of the PHBV and PHBV—GO/MXene composite membranes decreased with increasing pH. The pure PHBV membranes were more negative than the PHBV—GO/MXene composite membranes (Figure 5D). The GO/MXene-functionalized PHBV membranes had remarkably different surface charge features and became less negative owing to the decreasing of carboxyl groups. Most of the carboxyl groups in GO/MXene reacted with oxhydryl groups in PHBV, forming C-O-C bonds, which was confirmed by the FT-IR analysis results (Figure 5A). Due to progressive deprotonation, the ζ values of the PHBV and PHBV—GO/MXene composite membranes were less than zero. The isoelectric point (pI) values of PHBV appeared at 2.30. Moreover, the pI values of PHBV—GO/MXene 0.5% and PHBV—GO/MXene 1% composite membranes were 3.25 and 3.43, respectively. The remarkably different surface charge features of PHBV—GO/MXene composite membranes indicate that the incorporation of GO/MXene significantly enhanced the surface charge properties of PHBV. Some investigations have revealed that platelets are negatively charged [71,72]. GO/MXene addition reduces the electronegativity of PHBV (Figure 5D). Hence, the results of zeta potential analysis indicate that GO/MXene can enhance the adsorption capacity of the PHBV—GO/MXene composite membrane towards platelets. These results may suggest that this kind of composite material has good application prospects for haemostatic materials.

**Figure 6:**
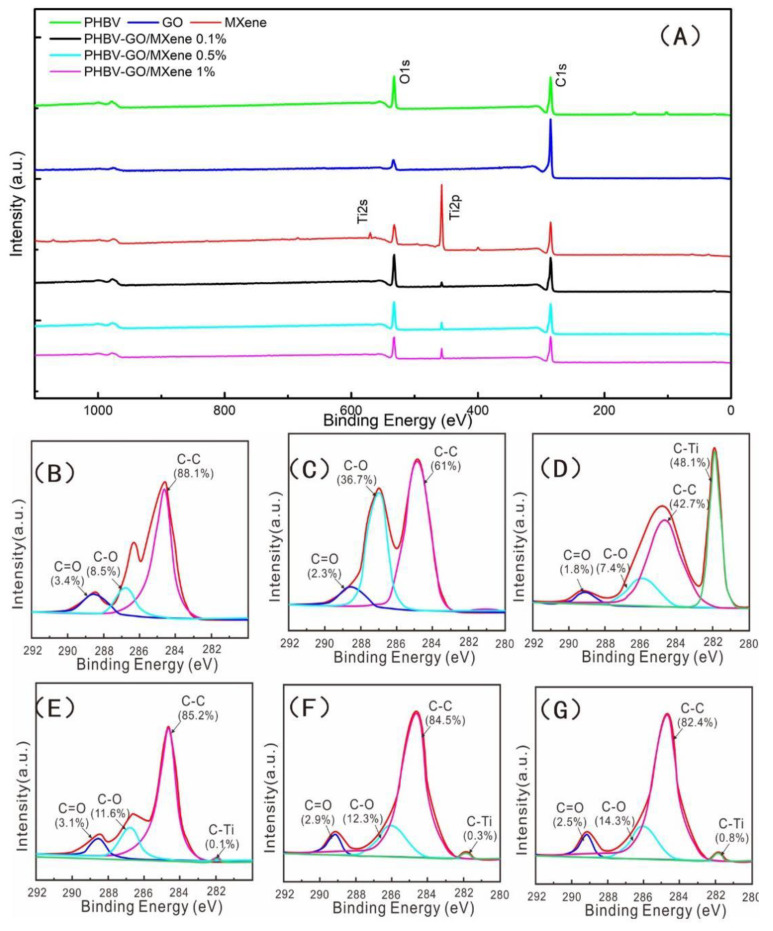
XPS spectrum (**A**) of PHBV, GO, MXene, PHBV—GO/MXene 0.1%, PHBV—GO/MXene 0.5%, and PHBV—GO/MXene 1% composite membranes. C1s XPS spectra of PHBV (**B**), GO (**C**), MXene (**D**), PHBV—GO/MXene 0.1% (**E**), PHBV—GO/MXene 0.5% (**F**), and PHBV—GO/MXene 1% (**G**) composite membranes.

To quantify the binding forms of oxygen, carbon, nitrogen, and titanium in the PHBV and PHBV—GO/MXene composite membranes, high resolution XPS spectra of O1s, C1s, Ti2p and Ti2s were deconvoluted using Gaussian-Lorentzian peaks [37,73,74]. The XPS results of the PHBV and PHBV—GO/MXene composite membranes are illustrated in Figure 6. The wide-scan XPS spectra showed sharp peaks of O1s and C1s (Figure 6A), which indicate that the pure PHBV membrane mainly contained elements of C and O. These results are consistent with the SEM-EDS results (Figure 2A and Figure S1). Further investigation illustrated that the intensities of the C=O, C-O, and C-C bonds were 3.4%, 8.5%, and 88.1%, respectively (Figure 6B). The C1s peak for GO was fitted to C=O, C-O, and C-C peaks (Figure 6C), which were similar to the PHBV results. However, the intensity of C-O in GO was higher than that in PHBV. Figure 6D shows that the C1s peak for MXene can be fitted to C=O, C-O, C-C, and C-Ti peaks (Figure 6D). The significant intensity of C=O and C-O confirmed the existence of hydrophilic oxygen-containing functional groups. The wide-scan XPS spectra showed that the PHBV—GO/MXene composite membrane contained elements of Ti introduced by MXene (Figure 6A). These results were also consistent with the results of SEM-EDS (Figure 2A and Figures S2–S4). The intensity of the C-O and C-Ti bonds increased with the increasing GO/MXene addition (Figure 6E–G), indicating that some GO and MXene were grafted onto the surface of the PHBV—GO/MXene composite membranes. In addition, the increase in C-O bonds was significantly higher than the summation of PHBV and GO/MXene, for the low GO/MXene addition amounts. Combining the high C-O peak in PHBV—GO/MXene and the low C-O peak in PHBV, GO, and MXene in FT-IR, the high intensity of C-O may suggest that GO was esterified with PHBV and the graft reaction occurred. These factors may be beneficial to toughen the PHBV—GO/MXene composite membranes.

### 3.3. Mechanical Property, Antibacterial Activity, and Platelet Adsorption

**Figure 7:**
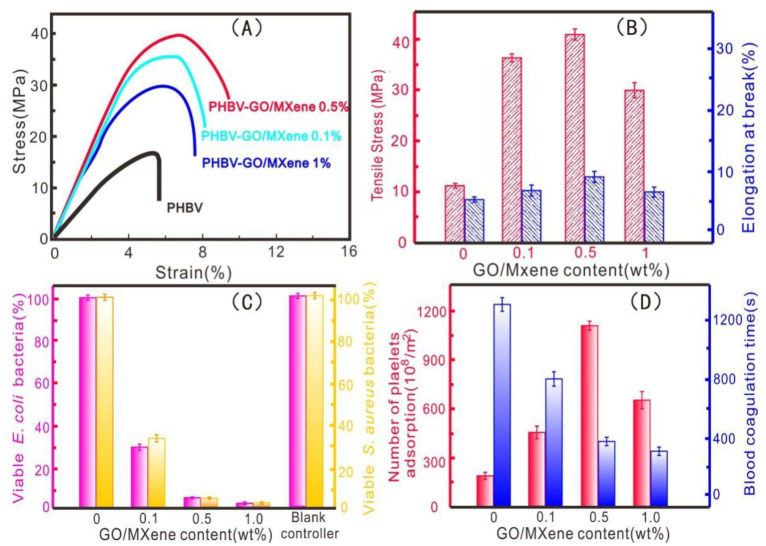
Stress-strain curves (**A**), elongation at break (**B**), antibacterial activity at 24 h and (**C**) platelet adsorption and blood coagulation time (**D**) of PHBV of PHBV, PHBV—GO/MXene 0.1%, PHBV—GO/MXene 0.5% and PHBV—GO/MXene 1% composite membranes.

The mechanical properties of the PHBV and PHBV—GO/MXene composite membranes were tested. The tensile strength and elongation at break were obtained, as shown in Figure 7A,B. The pure PHBV composite membranes exhibited low tensile stress (16 MPa) and low strain at break (4.6%) (Figure 7A,B). The tensile stress and strain at break are similar to those of commercial PHAs [75,76]. The tensile strengths of the PHBV—GO/MXene 0.1% and PHBV—GO/MXene 0.5% composite membranes were 36 MPa and 42 MPa, respectively, which are comparable to those of PHA/GO-g-LAQ 5 wt% films with 39.8 MPa tensile strength [4], higher than that of the PHA/ZnO 4 wt% nanocomposites, with 29.5 MPa tensile strength and PHA/nanofibrillated cellulose (NFC) nanocomposites 5 wt%, with a tensile strength of 34.4 MPa [75,76]. However, the tensile strength of the PHBV—GO/MXene composite membranes decreased from 42 MPa to 29 MPa as GO/MXene content increased from 0.5% to 1.0% (Figure 7A). This change may be triggered by the poor interfacial reaction between PHBV and GO/MXene (Figure 5A) and the aggregation effect of GO/MXene forming the fusiform structure, which also led to a decrease in elongation at break of the PHBV—GO/MXene 1.0% composite membranes. Good dispersion and esterification reactions may correspond to the increasing elongation at break of the PHBV—GO/MXene 0.1% and PHBV—GO/MXene 0.5% composite membranes (Figure 5B). These results confirmed that GO/MXene can greatly improve the tensile strength and elongation at break of PHBV.

*E. coli* and *S. aureus* bacteria are generally used to detect antibacterial activity against gram-negative and gram-positive bacteria [4,75,77,78]. The antimicrobial activity against *E. coli* and *S. aureus* bacteria of the PHBV and PHBV—GO/MXene composite membranes was detected. The results are shown in Figure 7C and Figure S4. After 24 h of incubation, the *E. coli* and *S. aureus* bacteria aggregated on the film surface and formed larger colonies on the PHBV composite membranes, which indicates that PHBV has a poor bactericidal ability (Figure 7C and Figure S5). Compared to the PHBV composite membranes, PHBV—GO/MXene 0.1% composite membranes showed good bacterial reduction in *E. coli* and *S. aureus* bacteria. The antibacterial activity of PHBV—GO/MXene 0.5% composite membranes reached 95% (Figure 7C and Figure S5). The antibacterial activity of PHBV—GO/MXene composite membranes increased from 95% to over 99% and the GO/MXene content increased from 0.5% to 1.0% (Figure 7C and Figure S5). These results suggest that PHBV—GO/MXene had a good antibacterial performance for the addition of GO/MXene. Although some studies reported that GO has no antibacterial activity [4,77], GO/MXene still showed excellent antibacterial properties in this investigation. These results may be due to the excellent antibacterial properties of MXene [48].

Generally, haemostatic capacity can be characterized by the number of platelets, adsorption and blood coagulation time [55]. The adsorption capacity towards platelet and blood coagulation of PHBV and PHBV—GO/MXene composite membranes are presented in Figure 7D. The pure PHBV composite membranes had a low adsorption capacity towards platelets and long blood coagulation times (Figure 7D). Compared to pure PHBV, PHBV—GO/MXene composite membranes had good platelet adsorption capacity. Among them, the PHBV—GO/MXene 0.5% composite membranes adsorbed 1143 ± 43 × 10^8^/m^2^, almost five times that of the PHBV composite membranes (Figure 7D). The good platelet-adsorption performance were consistent with the conclusions of BET and zeta potential, which indicate that PHBV—GO/MXene 0.5% composite membranes have good application prospects for haemostatic materials (Figure 4 and Figure 5D). Moreover, the blood coagulation time of the PHBV—GO/MXene 0.5% composite membranes was also short, at 379 ± 34 s, which was three times shorter than that of the PHBV composite membranes (Figure 7D). The blood coagulation time of the PHBV—GO/MXene 1.0% composite membranes was the fastest at 341 ± 28 s (Figure 7D). Therefore, the PHBV—GO/MXene 0.5% composite membranes showed the best procoagulant properties. These results also indicate that GO/MXene can significantly enhance the haemostatic properties of PHBV and PHBV—GO/MXene composite membranes, which may be valuable in wound healing applications.

Previous investigations have reported that platelet adsorption can be affected by many factors, such as surface properties [55]. Among them, hydrophobic surfaces can absorb more platelets compared to hydrophilic surfaces [50,79]. For the PHBV—GO/MXene composite membranes, the hydrophobic and rough surface may act as the active site for platelet adsorption, while the GO and MXene embedded in the fibres may act as hydrophilic active sites. Moreover, the GO and MXene may also react with PHBV and form more ester bond groups, which can further improve the tensile strength. Furthermore, due to the antibacterial properties of MXene, PHBV—GO/MXene composite membranes have good antibacterial properties. Therefore, we believe that the good properties of GO and MXene nanoparticles improve the mechanical properties, antimicrobial performance and platelet adsorption of PHBV. There are also some limitations of this study, due to the differences between rabbit and human platelets [80,81,82]. The 95–99% antibacterial activity of PHBV—GO/MXene composite membranes is also not a remarkable antibacterial performance. It is also necessary to improve the antibacterial ability of PHBV—GO/MXene composite membranes.

## 4. Conclusions

In this investigation, GO/MXene nanosheets were successfully prepared and grafted onto PHBV to obtain multifunctional, robust, and porous PHBV—GO/MXene composite membranes, with PVP acting as a pore-forming agent. The laminar structure and hydrophilicity of GO and MXene nanosheets gave PHBV—GO/MXene composite membranes superior hydrophilicity due to the presence of hydroxyl groups and terminal oxygen, which also provided functional site for the free radical polymerization of ester bonds between GO/MXene and PHBV. The interfacial embeddedness of GO/MXene nanosheets in the PHBV matrix significantly induced the crystallization behaviours of PHBV. Therefore, the mechanical properties, antimicrobial performance, and platelet adsorption were remarkably improved, which indicates that GO/MXene nanosheets contribute to enhancing the properties of PHBV. The tensile strength, platelet adsorption, and blood coagulation time of PHBV composite membranes were improved from 16 MPa, 213 ± 28 × 10^8^/m^2^, and 1312 ± 116 s to 42 MPa, 1143 ± 43 × 10^8^/m^2^, and 379 ± 34 s after incorporation of 0.5 wt% GO/MXene nanosheets. The antimicrobial performance also reached 95% for the PHBV—GO/MXene 0.5% composite membranes. These data supported that our investigation may provide a promising method for preparing facile and high-performance PHBV nanocomposites that could offer prosperous applications for multifunctional haemostatic materials.

## Figures and Tables

**Table 1:** The element composition and properties of PHBV and PHBV—GO/MXene composite membranes.

	PHBV	PHBV—GO/MXene 0.1%	PHBV—GO/MXene 0.5%	PHBV—GO/MXene 1%
Element components% (C/O/Ti) ^a^	79.03/20.97	77.26/21.82/0.07	75.22/23.05/0.28	75.63/23.38/0.75
Fibre diameter (FD) (μm) ^b^	4.32 ± 1.93	3.23 ± 2.06	3.16 ± 2.43	1.36 ± 2.06
Superficial area (SA) (m^2^ g^−1^) ^c^	16.75	/	45.51	24.46
Pore volume (ml g^−1^) ^d^	0.023	/	0.051	0.035
Dimension (D) ^e^	2.371	/	2.489	2.563
Isoelectric point (IEP)	4.23	/	4.02	3.74
Water contact angle (^o^) ^f^	130.0 ± 2.3	54.2 ± 3.1	62.2 ± 1.8	55.9 ± 2.8

^a^ detected by EDS. ^b^ based on tape measure in SEM images (Mean ± SD). ^c^ based on BET. ^d^ NLDFT. ^e^ based on BET. ^f^ Angle (Mean ± SD).

## Data Availability

Not applicable.

## Supplementary Materials

The following are available online at https://www.mdpi.com/article/10.3390/polym13213748/s1, Figure S1: The SEM-EDS mapping of PHBV composite membranes, Figure S2: The SEM-EDS mapping of PHBV—GO/MXene 0.1% composite membranes, Figure S3: The SEM-EDS mapping of PHBV—GO/MXene 0.5% composite membranes, Figure S4: The SEM-EDS mapping of PHBV—GO/MXene 1.0% composite membranes; Figure S5: Formation of bacteria colonies: antibacterial activity of PHBV, PHBV—GO/MXene 0.1%, PHBV—GO/MXene 0.5%, PHBV—GO/MXene 1%, and blank group composite membranes for *E. coli* and *S. aureus*, method of the antibacterial activity test and detail platelet adsorption experiments
