# Supplementary material for: Multifunctional, Robust, and Porous PHBV—GO/MXene Composite Membranes with Good Hydrophilicity, Antibacterial Activity, and Platelet Adsorption Performance
Source: Polymers (Basel). 2021 Oct 29;13(21):3748. doi: 10.3390/polym13213748 (PMC8588032; doi:10.3390/polym13213748)
Supplement: Supplementary file 1 [file polymers-13-03748-s001.zip › polymers-1412192-supplementary.pdf]

Multifunctional, Robust, and Porous PHBV-GO/MXene Composite Membranes with Superior Hydrophilia, Antibacterial Activity and Platelet Adsorbing Performances

Yuandong Wu[1,2], Weishaung Zheng[1,2], Yinan, Xiao[1,2], Yuanju Li[1,2], Xingru Zhang[1,2], Beining Du[1,2], Chen Lai[1,2], Yi Huang[1,2,3] and Liyuan Sheng[1,2*]

[1]Shenzhen Institute, Peking University, Shenzhen 518057, China
[2]PKU-HKUST ShenZhen-HonKong Institute, Shenzhen 518057, China
[3] School of Environmental Science and Engineering, Peking University, Beijing, 100871, China
* Correspondence: author (email: shengly@ier.org.cn)

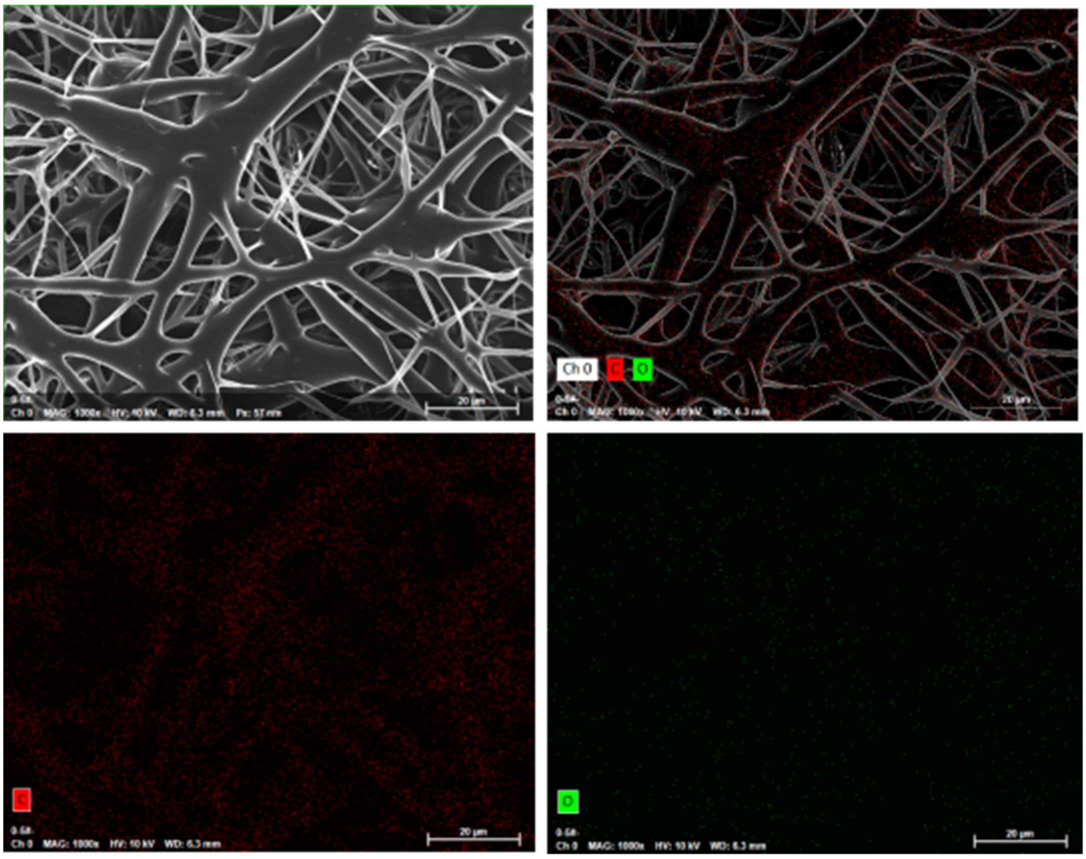

Figure S1. The SEM-EDS mapping of PHBV composite membranes.

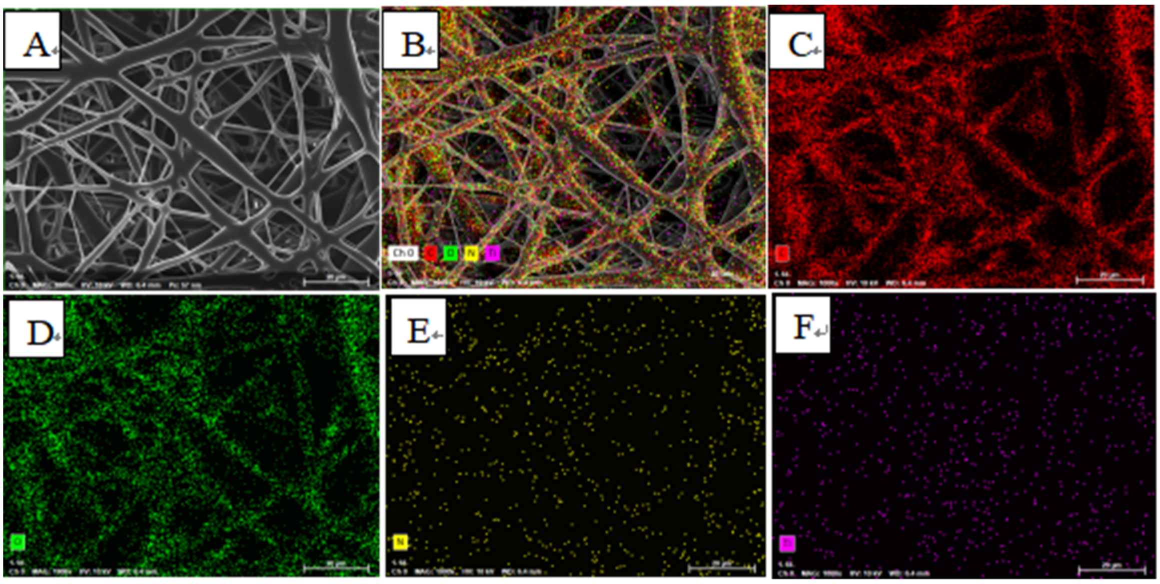

Figure S2. The SEM-EDS mapping of PHBV- GO/MXene 0.1% composite membranes.

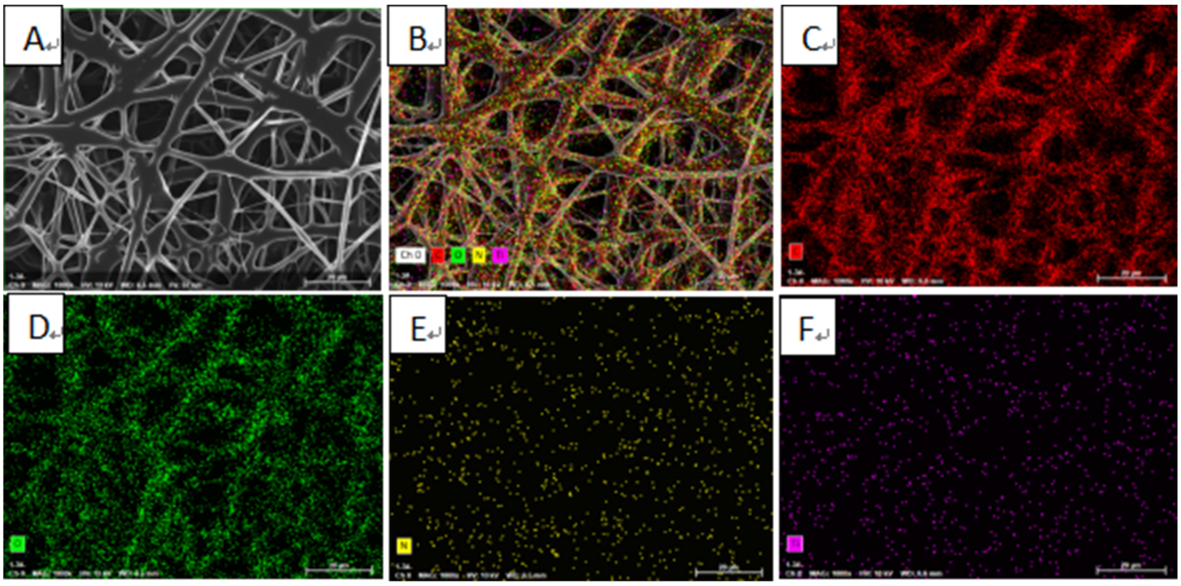

Figure S3. The SEM-EDS mapping of PHBV- GO/MXene 0.5% composite membranes.

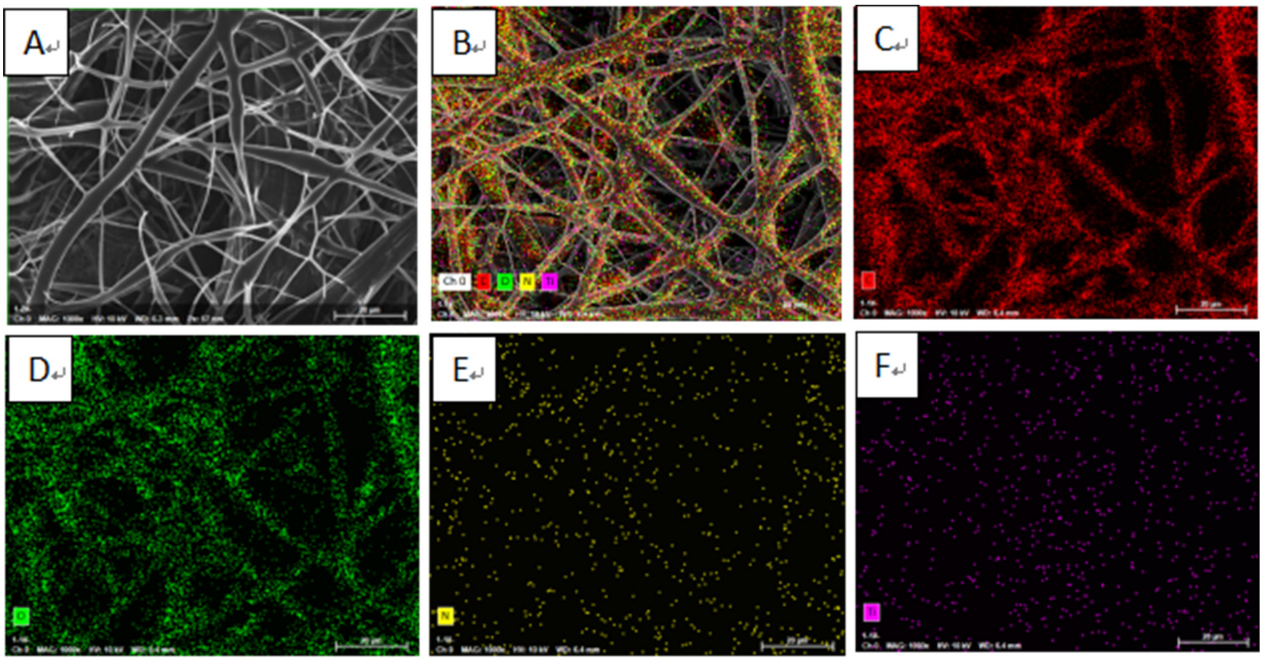

Figure S4. The SEM-EDS mapping of PHBV- GO/MXene 1.0% composite membranes.

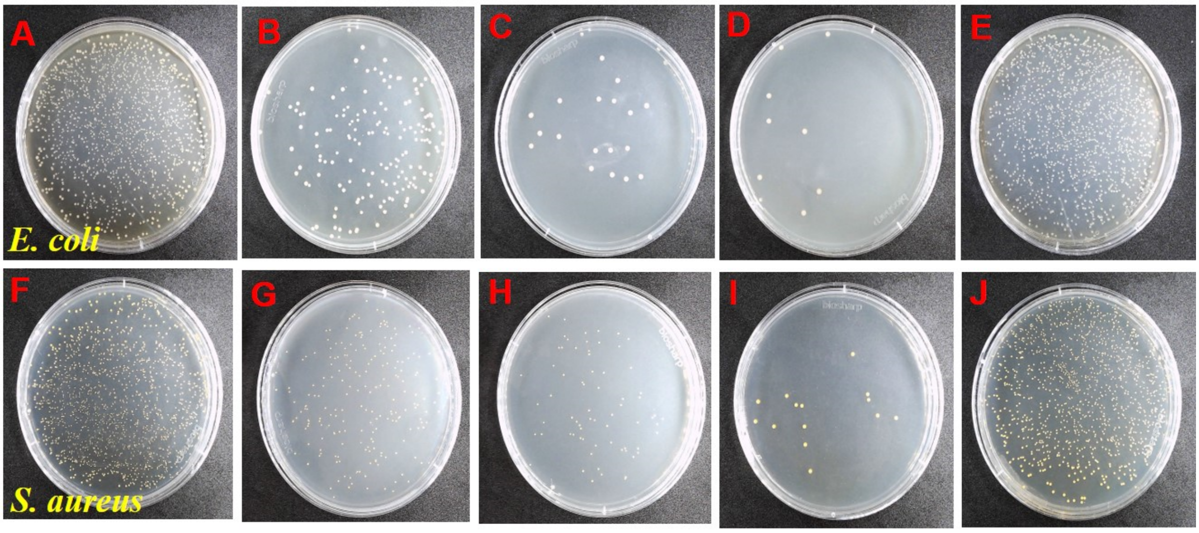

Figure S5. Formation of bacteria colonies: antibacterial activity of PHBV (A, F), PHBV-GO/MXene 0.1% (B, G), PHBV-GO/MXene 0.5% (C, H), PHBV-GO/MXene 1 % (D, I) and Blank group (E, J) composite membranes for *E. coli* and *S. aureus*.

Method of theantibacterial activity test

The antibacterial experiment of PHBV-MXene composite membranes is divided into 11 groups: LB solid medium blank control group; Escherichia coli + LB solid medium group; Staphylococcus aureus + LB solid medium group; PHBV+ Escherichia coli + LB solid medium Group; PHBV+Staphylococcus aureus+LB solid medium group; PHBV+0.1%MXene+E.coli+LB solid medium group; PHBV+0.5%MXene Staphylococcus aureus+LB solid medium group; PHBV+1%MXene+large intestine Bacillus+LB solid medium group; PHBV+0.1%MXene Staphylococcus aureus+LB solid medium group; PHBV+0.5%MXene+E.coli+LB solid medium group; PHBV+1%MXene Staphylococcus aureus+LB solid group. Each group had three replicates.

After the experiment grouping is completed, prepare LB solid medium and liquid medium. The composition of Luria broth (LB)solid medium is: 10 g/L peptone, 10 g/L NaCl, 5.0 g/L beef extract and 20% agar powder. A total of 11*3=33 plates are required. According to each 90mm plate pour 20ml of liquid medium, a total of 33*20=660ml LB

solid medium is required, which is configured at 0.7L. The LB liquid medium is prepared according to the above method, and the prepared LB solid and medium are placed in a sterilization pot and sterilized at 120°C for 30 minutes. LB liquid medium was inoculated with Escherichia coli and Staphylococcus aureus. After the inoculation was completed, it was placed on a shaker at 37°C and 200 rpm for culture. Escherichia coli was cultured for 16 hours, and Staphylococcus aureus was cultured for 24 hours. At the same time, the sterilized LB solid medium is still in a liquid state when the temperature is higher than 40°C and can be poured into the plate. After it is cooled for 24 hours, the LB solid medium plate will be formed. After the above steps are completed, sterilization experiments are carried out in groups according to the experiment.

PHBV is 10mg, and MXene is respectively 10μg, 50μg and 100μg. The bacterial solution is 10μl (10-8CFU/ml). According to the grouping, use the coating rod to complete the above experiment. Pay attention to aseptic operation during the experiment. To finish, burn the coating rod on an alcohol lamp for 30 seconds, and wait for it to cool before applying the next flat plate. After coating the 33 plates, place them in a constant temperature incubator at 37°C for 24 hours. After that, observe the number of colonies on the plates, and take photos of 33 plates one by one and keep files.

The antibacterial activity against *E. coli* (α) and *S. aureus* (β) of PHBV and PHBV-GO/MXene composite membranes were calculated using equations (1) and (2), respectively.

$$\alpha = \frac{C_t}{C_0} \times 100\% \quad (1)$$

$$\beta = \frac{D_t}{D_0} \times 100\% \quad (2)$$

where C_0 and C_t are the colony numbers of *E. coli* in blank group and experimental groups, respectively. D_t represents the colony numbers of *S. aureus* in experimental groups, and D_0 is colony numbers of *S. aureus* in blank group.

Platelet Adsorption experiments:

The hemostatic properties of PHBV and PHBV-GO/MXene composite membranes were evaluated by platelet adsorption and blood coagulation. The protocol was approved by the ethics committee at the Chinese Academy of Sciences. Fresh blood obtained from a healthy rabbit was mixed with 3.8% anticoagulant sodium citrate (2:8). To prepare platelet-rich plasma (PRP), the blood samples were centrifuged at 1, 500 rpm for 10 min at 4 ° C. The copolymer membranes in glass dishes were sterilized with 75% ethanol, rinsed with PBS three times, and equilibrated in PBS overnight. After being warmed to 37 ° C, 1 mL of PRP was added to the tested membranes and incubated at 37 ° C for 1 h. Films were then rinsed three times with PBS to remove weakly absorbed platelets from the membranes surface. The platelet-attached membranes were fixed, dehydrated, dried, and gold sputtered for examination. Platelet were detected before and after adsorption using platelet analyzer. Each copolymer had three parallel membranes. The results are reported as the average number of adhered platelets per square meter of surface. Blood coagulation time was measured according to a previously published method (Wu et al., 2008).

References

Wu L P, You M, Wang D, et al. Fabrication of carbon nanotube (CNT)/poly (3-hydroxybutyrate-co-3-hydroxyhexanoate) (PHBHHx) nanocomposite films for human mesenchymal stem cell (hMSC) differentiation. Polymer chemistry, 2013, 4(16): 4490-4498.
